# Cost-effectiveness of gargling for the prevention of upper respiratory tract infections

**DOI:** 10.1186/1472-6963-8-258

**Published:** 2008-12-16

**Authors:** Michi Sakai, Takuro Shimbo, Kazumi Omata, Yoshimitsu Takahashi, Kazunari Satomura, Tetsuhisa Kitamura, Takashi Kawamura, Hisamitsu Baba, Masaharu Yoshihara, Hiroshi Itoh, the Great Cold Investigators-I

**Affiliations:** 1 Department of Clinical Research and Informatics, Research Institute, International Medical Center of Japan, Tokyo, Japan; 2 Department of Epidemiology and Healthcare Research, Kyoto University School of Public Health, Kyoto, Japan; 3 Center for Health Service, Outcomes Research and Development, Japan (CHORD-J), Tokyo, Japan; 4 Department of Public Health and International Health, Kyoto University School of Public Health, Kyoto, Japan; 5 Osaka Saiseikai Senri Hospital, Osaka, Japan; 6 Department of Preventive Services, Kyoto University Health Service, Kyoto, Japan; 7 Medical Center for Student Health & Department of Biosignal Pathophysiology, Graduate School of Medicine, Kobe University, Kobe, Japan; 8 Health Service Center, Hiroshima University, Hiroshima, Japan; 9 Ritsumeikan University, Kyoto, Japan

## Abstract

**Background:**

In Japan, gargling is a generally accepted way of preventing upper respiratory tract infection (URTI). The effectiveness of gargling for preventing URTI has been shown in a randomized controlled trial that compared incidences of URTI between gargling and control groups. From the perspective of the third-party payer, gargling is dominant due to the fact that the costs of gargling are borne by the participant. However, the cost-effectiveness of gargling from a societal perspective should be considered. In this study, economic evaluation alongside a randomized controlled trial was performed to evaluate the cost-effectiveness of gargling for preventing URTI from a societal perspective.

**Methods:**

Among participants in the gargling trial, 122 water-gargling and 130 control subjects were involved in the economic analysis. Sixty-day cumulative follow-up costs and effectiveness measured by quality-adjusted life days (QALD) were compared between groups on an intention-to-treat basis. Incremental cost-effectiveness ratio (ICER) was converted to dollars per quality-adjusted life years (QALY). The 95% confidence interval (95%CI) and probability of gargling being cost-effective were estimated by bootstrapping.

**Results:**

After 60 days, QALD was increased by 0.43 and costs were $37.1 higher in the gargling group than in the control group. ICER of the gargling group was $31,800/QALY (95%CI, $1,900–$248,100). Although this resembles many acceptable forms of medical intervention, including URTI preventive measures such as influenza vaccination, the broad confidence interval indicates uncertainty surrounding our results. In addition, one-way sensitivity analysis also indicated that careful evaluation is required for the cost of gargling and the utility of moderate URTI. The major limitation of this study was that this trial was conducted in winter, at a time when URTI is prevalent. Care must be taken when applying the results to a season when URTI is not prevalent, since the ICER will increase due to decreases in incidence.

**Conclusion:**

This study suggests gargling as a cost-effective preventive strategy for URTI that is acceptable from perspectives of both the third-party payer and society.

## Background

Prevention of upper respiratory tract infection (URTI) represents a major public health issue. An average of 2.5 URTI episodes are reportedly experienced annually in the United States[1,2]. In Japan, 4.02% of physician visits are due to URTI, and the number of patients who consult physicians due to URTI has been estimated as 223 of 100,000 in a day[3]. Uniquely in Japan, gargling is generally accepted and strongly recommended as a preventive measure for URTI. In addition to hand washing and the wearing of masks, the current guidelines for dealing with pandemic influenza in Japan also recommend gargling as a preventive measure[4].

Although the evidence for URTI prevention by gargling is limited, the effectiveness of gargling for preventing URTI among healthy people was shown in a randomized controlled trial that compared incidences of URTI between gargling and control groups[5]. This trial noted a 36% decrease in the incidence of URTI with water gargling.

In Japan, annual health care expenditures associated with acute URTI, including hospital fees and prescription medicines, total around US$5 billion[5]. A reduction in URTI incidence by up to 36% with water gargling would equate to a saving of approximately US$2 billion in annual health care costs[5]. From the perspective of patients, gargling is somewhat time-consuming, but can prevent about one-third of URTI cases. The decision on whether to gargle is up to the individual. From the perspective of the third-party payer, gargling is a dominant preventive strategy due to the fact that the opportunity cost of gargling is imposed on the participant. However, the cost-effectiveness of gargling from a societal perspective should be fully considered. A trade-off exists between effectiveness for reducing the incidence of URTI and the opportunity costs incurred. An economic evaluation was therefore performed alongside a randomized controlled trial to evaluate the cost-effectiveness of gargling for preventing URTI from a societal perspective.

## Methods

### Setting and patients

From December 2002 through January 2003, healthy adult volunteers aged between 18 and 65 years were recruited and randomly assigned to a water gargling group, povidone-iodine gargling group or control group, as described in detail previously[5]. A total of 387 subjects participated in the study. Excluded from analysis were 2 subjects who displayed URTI on the first day of intervention, and 1 subject who did not write in the diary at all (follow-up, 99%). Included in the analysis were a total of 384 patients, with 122 patients in the water-gargling group, 132 patients in the povidone/iodine-gargling group, and 130 patients in the control group. Baseline characteristics and outcomes of gargling and control groups are shown in Table 1.

**Table 1:** Characteristics and outcomes of the RCT subjects

	Gargling (n = 122)	Control (n = 130)
Baseline characteristics		
	
Gender (male/female)	39/83	43/87

Age (mean)	34.7	36.2

Anti-influenza vaccination (%)	14.3	19.2

Frequency of URTIs in preceding year	14/71/36	16/78/36
(0/1–2/> = 3 times)*		

Outcomes of the trial		
Infected cases (%)**	30.1	40.8
Incidence rate per 60 person-days	0.34	0.52
Duration of illness (days) ***	88	156

* Data missing for 1 participant.

** Estimated by Kaplan-Meier.

*** Duration of moderate or severe URTI

Gargling groups were instructed to gargle with approximately 20 ml of water or povidone-iodine for about 15 s, 3 times/day. Control groups were instructed to retain previous gargling habits. The primary outcome measure was first URTI incidence within 60 days. Sample size of the trial was calculated at a power level of 0.90 and a significance level of 0.05. Analyses were performed on an intention-to-treat basis.

Frequency of gargling and presence of various URTI complaints in all subjects were also assessed using the self-administered record (gargling diary). All URTI complaints, such as nasal symptoms, pharyngeal symptoms, bronchial symptoms, pharyngeal symptoms, bronchial symptoms and general symptoms were recorded and classified by each subject into 4 grades as none, mild, moderate or severe, according to Jackson methods[6]. "Mild" was defined as being unaware of the symptom when busy, "moderate" as always feeling discomfort, and "severe" as having difficulties in completing the usual activities of daily living. Subjects who developed URTI were asked to continue completing the gargling diary for 1 week after onset of URTI symptoms to confirm the incidence and severity of URTI.

No subjects assigned to the water-gargling group skipped gargling, while 36 subjects (28%) in the control group did not gargle at all. Compared to 50 subjects (40.8% by Kaplan-Meier estimation) in the control group, 34 subjects (30.1%) in the water-gargling group (p = 0.044) and 46 subjects (37.2%) in the povidone/iodine-gargling group (p = 0.59) had developed URTI as of day 60. Incidences were lower in water-gargling subjects (0.34 episodes/60 person-days) and povidone/iodine-gargling subjects (0.48 episodes/60 person-days) than in controls (0.52 episodes/60 person-days), and rate ratios compared to controls were 0.64 (95% confidence interval (95%CI), 0.42–0.99) and 0.89 (95%CI, 0.60–1.33), respectively. In the present study, the cost and effectiveness of water gargling were determined by comparison with the control group. All study protocols were approved by the ethics committee of Kyoto University.

### Costs of care

The 60-day cumulative follow-up costs for all trial participants were estimated from a societal perspective. All costs were converted into US dollars according to Purchasing Power Parities in 2005[7], with a dollar considered equivalent to about 128 Japanese yen.

Costs of gargling, physician consultations due to URTI, medications to treat URTI, and lost productivity due to severe URTI were estimated (Table 1). Costs of gargling were estimated as the opportunity costs of the time required for gargling by multiplying the time to complete a single session of gargling, the frequency of gargling in each group, and the mean wage of Japanese workers[8]. Time to complete a single session of gargling, including going to and returning from the washroom, was determined based on 12 individuals who were not participants in this trial, with gargling considered to require an average of 71 s.

The cost of a physician consultation was estimated by multiplying the proportion of subjects who visited physicians due to URTI and the costs involved in such visits. The proportion of subjects who visited physicians was obtained from the literature[9], since this information was not recorded in the gargling trial. The cost of physician consultation was estimated from the sum of the first visit fee, the cost of the time required for the consultation, and the transportation fee. The latter two costs were obtained from the Patients' Behavior Survey[10], with time converted to a cost based on national wage and labor time statistics[11]. The daily cost of medicine was estimated based on the Survey for Individual Medical Procedures[11]. The cost of lost productivity was estimated assuming that patients with severe URTI were unable to work all day. All costs are expressed in 2005 costs.

### Effectiveness

Effectiveness was measured in quality-adjusted life days (QALD). Utility was assigned to each day according to the duration and severity of URTI, with the 60-day cumulative QALD gained calculated for each strategy. Utilities in severe and moderate URTI were considered to be decreased. These utilities were derived from a previous study that measured utility in influenza[12]. Health states in severe URTI were estimated as the average utility from day 1 to day 3 of influenza, and in moderate URTI as the average utility from day 4 to day 7 of influenza (Table 1).

### Analysis

Differences in 60-day cumulative follow-up costs and effectiveness between gargling and control groups were compared on an intention-to-treat basis, and the incremental cost-effectiveness ratio (ICER) was derived.

In this trial, the endpoint was the onset of URTI, and affected patients were censored. The average cost and effectiveness for each day were therefore estimated based on those from the number of participants observed on each day, then the 60-day average cost and effectiveness were summed to calculate differences between groups. Censoring in the cost estimation was adjusted according to the methods described by Lin et al[13]. ICER was calculated from differences between gargling and control groups in 60-day cumulative costs and QALD. The ICER unit was converted to quality-adjusted life years (QALY) for convenience. The 95%CIs were calculated using the bootstrap method, using 5000 resamplings with replacement of participants in this trial.

### Sensitivity analysis

One-way sensitivity analyses were performed for all costs and utilities within ± 50% ranges to assess the effects of uncertainty related to parameter estimates. Further two-way sensitivity analyses were applied to evaluate combinations of gargling cost and utility of moderate URTI.

## Results

Of the 384 participants in the gargling trial, 122 subjects assigned to water-gargling and 130 subjects assigned to the control group were included in the economic analysis. Baseline characteristics of the two groups are shown in Table 2. During the 60-day follow-up, incidence of the first URTI was 0.26 episodes/30 person-days in the control group and 0.17 episodes/30 person-days in the water-gargling group[5].

**Table 2:** Estimated costs and utility

**Variable**	**Value**
**Cost per day**	
Gargling (once)	$0.4
Visiting physicians (once)*	$47.9
Medicine (per day)	$2.0
Lost productivity due to severe URI (per day)	$97.7

**Utility**	
Moderate URI	0.63
Severe URI	0.24

* Only 36% of those who developed URI were assumed to visit a physician.

### Estimated costs and effectiveness after 60 days (Table 3)

**Table 3:** Results of cost effectiveness analysis

	**Cost($)**	**Incremental** cost (95%CI)**	**Effectiveness** **(QALD)**	**Incremental** **effectiveness (95%CI)**	**ICER($/QALY)** (95%CI)**
**Gargling**					
Cost of gargling	80.4	62.6			
Cost of URTI	24.9	-25.5			
**Total**	**105.3**	**37.1 ** **(7.4–65.4)**	**59.52**	**0.43 ** **(0.07–0.80)**	**31,800 ** **(1,877–248,095)**

**Control**					
Cost of gargling	17.8				
Cost of URTI	50.4				
**Total**	**68.2**		**59.10**		

QALD = quality adjusted life days.

ICER = incremental cost-effectiveness ratio.

**95% confidence intervals calculated by the bootstrap method.

The 60-day cumulative follow-up costs were estimated at $105.3 for the gargling group and $68.2 for the control group, respectively. Difference between the groups was $37.1 (95%CI, $7.40–$65.40). The costs of gargling for each group were $80.40 in the gargling group and $17.80 in the control group, representing a $62.6 increase in the gargling group. The costs of URTI were $24.9 in the gargling group and $50.4 in the control group. Cost was thus $25.50 lower in the gargling group. The 60-day QALD was 59.52 in the gargling group and 59.10 in the control group, showing that QALD was greater by 0.43 (95%CI, 0.07–0.80) in the gargling group (Table 3).

### Cost-effectiveness analysis (Table 3)

**Figure 1:**
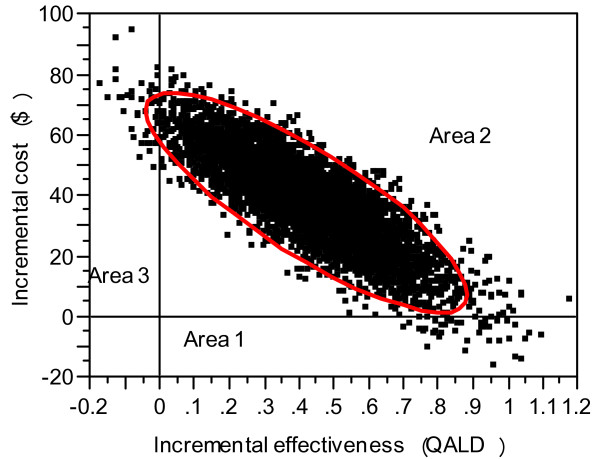
**Scatter plot of simulated mean cost and effect differences in 60 days.** Five thousand bootstrap samplings were used for the incremental cost and effectiveness of the gargling group compared to the control group. The plot indicates that 0.9% of all cases are located in area 1 indicating that  gargling is dominant, 98.2% of total cases are located in area 2 indicating that gargling is more costly and effective than control, and 0.9% of all cases are located in area 3 indicating that gargling is dominated by control.

**Figure 2:**
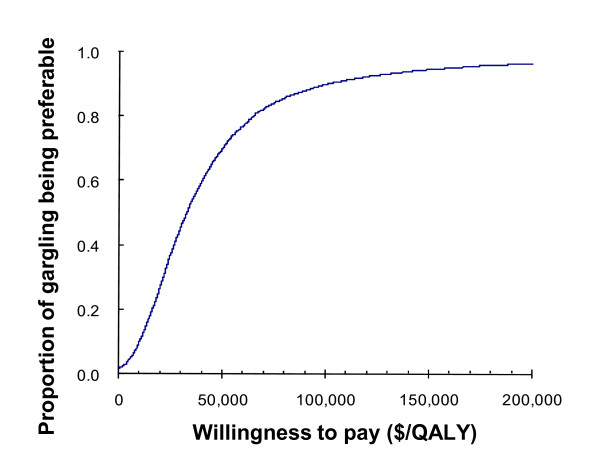
**Acceptability curve**. The curve indicates the probability of gargling being preferable to the control for potential maximum amounts that a decision-maker is willing to pay for an additional increase in QALY. WTP, willingness to pay.

The incremental cost per QALY gained associated with gargling was $31,800 (95%CI, $1,900–$248,100). Bootstrapped estimates of the incremental costs and incremental QALD are shown in Figure 1 using the cost-effectiveness plane. Figure 2 shows that, given a willingness-to-pay threshold of $50,000/QALY, the probability of gargling being cost-effective compared with control is 69.8%. If the threshold is increased to $100,000, then the probability increases to 89.9%.

### Sensitivity analyses (Table 4)

**Table 4:** Results of sensitivity analysis

	**Estimated costs and utilities**			ICER ($/QALY)*		
	**-50%**	**Baseline**	**+50%**	**-50%**	**Baseline**	**+50%**

**Costs ($)**						
Gargling (one time)	0.2	0.4	0.6	5,000	31,800	58,600
Physician consultation because of URTI	8.6	17.2	25.9	33,900	31,800	29,600
Medication to treat URTI	0.6	1.2	1.8	31,900	31,800	31,700
Lost productivity due to severe URTI	48.9	97.7	146.6	41,400	31,800	22,100

**Utility**						
Utility in moderate influenza	0.32	0.63	0.95	21,000	31,800	64,800
Utility in severe influenza	0.12	0.24	0.36	29,900	31,800	33,900

ICER = incremental cost-effectiveness ratio.

*ICERs for each group are rounded to the nearest $100.

**Figure 3:**
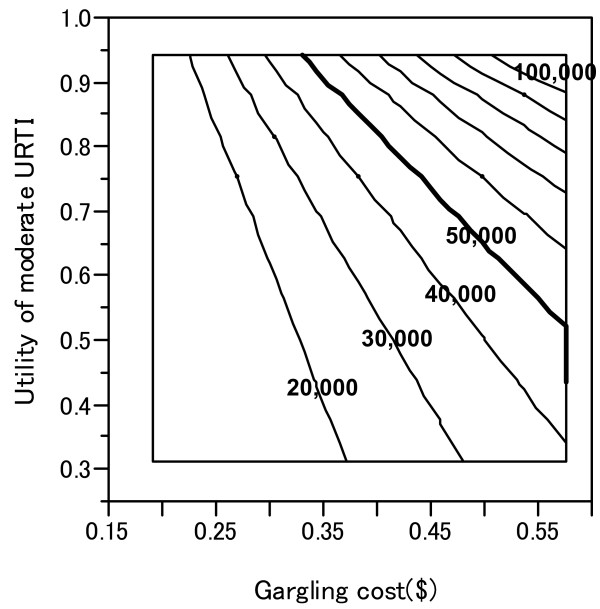
**Two-way sensitivity analysis of two factors: gargling cost and utility of moderate URTI.** Lines indicate the incremental cost effectiveness ratio ($/QALY) for gargling. The thick line indicates 50,000 $/QALY.

One-way sensitivity analysis showed that the ICER of gargling is highly sensitive to the cost of gargling and the utility of moderate influenza (Table 4). These 2 critical factors were studied further using two-way sensitivity analysis. Figure 3 shows the combination of gargling cost and utility of moderate URTI.

ICER for gargling varied from $5,000 to $58,600 when the cost of gargling ranged from $0.2 to $0.6. In addition, ICER varied from $21,000 to $64,800 when the utility of moderate influenza ranged from 0.32 to 0.95. ICER of gargling did not exceed $50,000/QALY in sensitivity analyses involving the following variables: cost of physician consultations due to URTI; cost of medications to treat URTI; and utility of severe influenza.

## Discussion

Although several limitations are inherent to performing an economic analysis alongside a randomized trial[14], this approach allowed quantification of the cost-effectiveness of gargling. Gargling generated a 0.43 increase in QALD and $37.1 higher costs compared with the control group. Although gargling generated a higher QALD by preventing URTI, the daily cost of gargling exceeded the cost of the URTI saved by gargling. ICER of the gargling group was $31,800/QALY (95%CI, $1,900–$248,100). This is similar to many acceptable forms of medical intervention, including URTI preventive methods such as influenza vaccination[12,15,16]. Although ICER of gargling was within the range of acceptable forms of URTI preventive methods such as influenza vaccination[12,15,16], the broad confidence interval indicates uncertainty surrounding our results. In addition, one-way sensitivity analysis showed that the cost of gargling and the utility of moderate URTI exerted a large impact on the cost-effectiveness of gargling. Careful evaluation is thus required for those variables.

We estimated the cost of gargling based on the average wage of Japanese workers based on the assumption that patients lost productivity due to gargling. If the impact on productivity is minimized and the cost of gargling can be maintained at lower than $0.16 (lower than the lower limit of the cost used in sensitivity analysis) gargling will be dominant.

The cost effectiveness of gargling also depends on how effectively it can reduce the incidence of influenza-like illnesses (ILI). The gargling trial was designed to evaluate the effectiveness of gargling for preventing URTI among healthy individuals, and therefore excluded ILI. Further analysis focusing on ILI was subsequently performed using the same data set[17]. Although no statistical significance was achieved due to the small number of ILI, analysis indicated a tendency toward decreased incidence of ILI with water gargling (hazard ratio, 0.75; 95%CI, 0.32–1.72). If the effectiveness of gargling in preventing ILI were to be demonstrated in a further study involving a large sample, the cost-effectiveness of gargling would be improved due to decreases in the number of patients suffering from complications of ILI and decreased use of oseltamivir.

The major limitation of our study was that this trial was conducted in winter, the season of maximum URTI prevalence. Care must therefore be taken when applying our results to seasons in which URTI is less prevalent, since the ICER will increase with a lower URTI incidence. Second, estimated costs for URTI, particularly for physician consultations resulting from URTI, were based on the assumption that the proportion of patients who visit clinics is 36%[9]. We examined the impact of variability of costs for URTI with one-way sensitivity analysis and showed the variability did not significantly affect the result. Finally, we were unable to estimate all opportunity costs, such as time required for dedicated trips to the washroom to gargle, as no precise data were available.

## Conclusion

In conclusion, the present study suggests that gargling has potential as a cost-effective preventive strategy for URTI that is acceptable from both third-party payer and societal perspectives. However, careful consideration of the uncertainties surrounding the estimation of ICER for gargling is required.

## Competing interests

The authors declare that they have no competing interests.

## Authors' contributions

MS drafted the manuscript and helped with data analysis. TS analyzed the cost effectiveness. KO and YT helped with data analysis. KS and TK conceived of this randomized controlled trial, designed the protocol, enrolled participants, and participated in data collection. TK managed the whole project as chief investigator. HB, MY, and HI contributed to the enrollment of patients. The Great Cold Investigators worked very well as a team during the study period.

All authors have read and approved the final manuscript.

## Pre-publication history

The pre-publication history for this paper can be accessed here:


